# Effect of Tea Polyphenols on Lipid Peroxidation and Antioxidant Activity of Litchi (*Litchi chinensis* Sonn.) Fruit during Cold Storage

**DOI:** 10.3390/molecules191016837

**Published:** 2014-10-20

**Authors:** Wenrong Chen, Zhenzhen Zhang, Yanwen Shen, Xuewu Duan, Yuemin Jiang

**Affiliations:** 1 South China Botanical Garden, Chinese Academy of Sciences, Guangzhou 510650, China; E-Mails: cwr@zjnu.cn (W.C.); xwduan@scbg.ac.cn (X.D.); 2 College of Chemistry and Life Science, Zhejiang Normal University, Jinhua 321004, China; E-Mail: shenyanwen2014@sina.cn; 3 Dinghai Forestry Workstation, Zhoushan 316000, China; E-Mail: zhangzhenzhen1983@sina.com

**Keywords:** litchi, tea polyphenols, reactive oxygen species (ROS), lipid peroxidation, antioxidative activity

## Abstract

To understand the potential of application of tea polyphenols to the shelf life extension and quality maintenance of litchi (*Litchi chinensis* Sonn.) fruit, the fruits were dipped into a solution of 1% tea phenols for 5 min before cold storage at 4 °C. Changes in browning index, contents of anthocyanins and phenolic compounds, superoxide dismutase (SOD) and peroxidase (POD) activities, O_2_^.−^ production rate and H_2_O_2_ content, levels of relative leakage rate and lipid peroxidation, and 1,1-diphenyl-2-picrylhydrazyl (DPPH) radical scavenging activity were measured after 0, 10, 20 and 30 days of cold storage. The results showed that application of tea polyphenols markedly delayed pericarp browning, alleviated the decreases in contents of total soluble solids (TSS) and ascorbic acid, and maintained relatively high levels of total phenolics and anthocyanins of litchi fruit after 30 days of cold storage. Meanwhile, the treatment reduced the increases in relative leakage rate and lipid peroxidation content, delayed the increases in both O_2_^.−^ production rate and H_2_O_2_ contents, and increased SOD activity but reduced POD activity throughout this storage period. These data indicated that the delayed pericarp browning of litchi fruit by the treatment with tea polyphenols could be due to enhanced antioxidant capability, reduced accumulations of reactive oxygen species and lipid peroxidation, and improved membrane integrity.

## 1. Introduction

Litchi (*Litchi chinensis* Sonn.) is a tropical to subtropical fruit with high commercial value, due to its white translucent aril and attractive red peel color [1]. However, pericarp browning and decay are identified as major constraints affecting the quality of litchi fruit after harvest, which reduced greatly market value [2]. Litchi pericarp browning is mainly attributed to the oxidation of polyphenols and/or the degradation of anthocyanins by polyphenol oxidase (PPO) and peroxidase (POD), resulting in the formation of polymeric brown byproducts [1,3,4]. The oxidation of phenolics and degradation of anthocyanins could be due to accumulated reactive oxygen species (ROS) [5]. On the other hand, the presence of antioxidant defense systems can protect plants from ROS damage [6]. Among the antioxidant defense system, superoxide dismutase (SOD) can scavenge superoxide anion (O_2_^.−^) radicals into H_2_O_2_, while H_2_O_2_ can further be converted into water by catalase [6]. It was found that the litchi anthocyanins can be degraded markedly in the presence of H_2_O_2_ or hydroxyl radical, to which the pericarp browning of litchi fruit during storage was attributed [5]. Therefore, to reduce ROS production, delay or inhibit enzymatic oxidation, and inhibit the oxidation of phenolics and the degradation of anthocyanins could be an important means to extend the storage life and maintain the quality of harvested litchi fruit.

Currently, pericarp browning of harvested litchi fruit can be inhibited effectively by sulphur dioxide (SO_2_) fumigation. Increasing concerns have arisen however about the SO_2_ residues present in the fruit. Recent researches reveal that application of antioxidative compounds may extend the storage life of harvested fruit. For example, both endogenous and exogenous phenolics can inhibit the anthocyanin degradation caused by polyphenol oxidase in litchi fruit [7]. The use of glutathione combined with citric acids were also found to give a good control of the browning in litchi fruit during storage [8]; The use of anthocyanins extracted from seed coats of black beans markedly delayed pericarp browning of litchi fruit during storage [4]. Thus, it can be speculated that the application of exogenous polyphenols may inhibit fruit browning through the inhibition of polyphenol oxidase, maintenance of antioxidant ability and reduction of lipid peroxidation.

**Figure 1:**
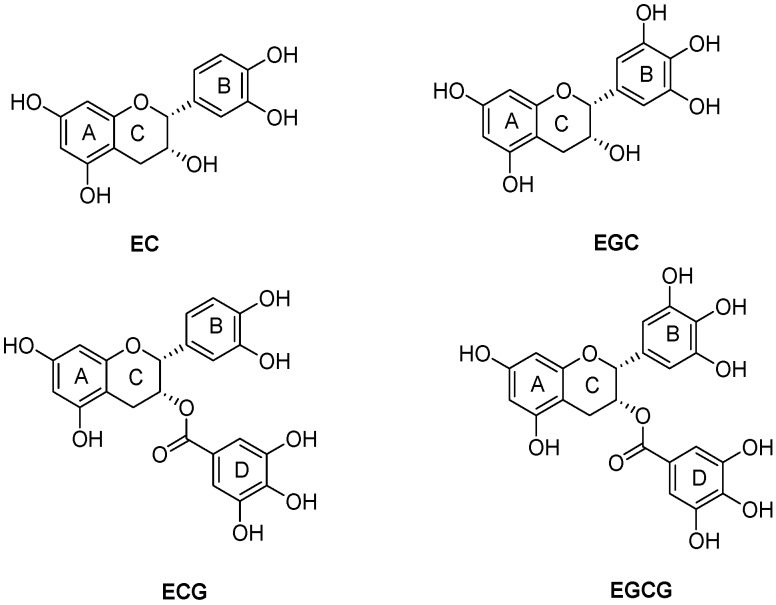
The chemical structures of major green tea polyphenols: The polyphenols that exist in green tea mainly consist of (+)-epicatechin (EC), (−)-epigallocatechin (EGC), (−)-epicatechin gallate (ECG), and (−)-epigallocatechin gallate (EGCG).

Tea polyphenols are phenolic compounds obtained from tea leaves, which mainly consist of (+)-epicatechin (EC), (−)-epigallocatechin (EGC), (−)-epicatechin gallate (ECG), and (−)-epigallocatechin gallate (EGCG) (Figure 1) [9]. Emerging evidence has placed considerable emphasis on the antioxidative properties of tea polyphenols. The formation of ROS such as superoxide radical, singlet oxygen, hydroxyl ROS, nitric oxide, nitrogen dioxide, and peroxynitrite is found to be decreased by the scavenging ability of tea polyphenols. However, no researches have reported that tea polyphenols have the capacity to inhibit the activity of polyphenol oxidase or can be applied to retard the pericarp browning of the fruits.

The objective of this study was to investigate the effect of tea polyphenols on pericarp browning and the incidence of decay of harvested litchi fruit during storage. Changes in browning index, contents of anthocyanins and total phenols, superoxide dismutase (SOD) and peroxidase (POD) activities, O_2_^.−^ production rate and H_2_O_2_ content, levels of relative leakage rate and lipid peroxidation, and 1,1-diphenyl-2-picrylhydrazyl (DPPH) radical scavenging activity were measured to understand better the roles of tea polyphenols in controlling pericarp browning of harvested litchi fruit. The study could help develop further commercial postharvest handling procedures for quality maintenance and shelf life extension of litchi fruit during storage and transportation.

## 2. Results

### 2.1. The Change in Browning Index

**Figure 2:**
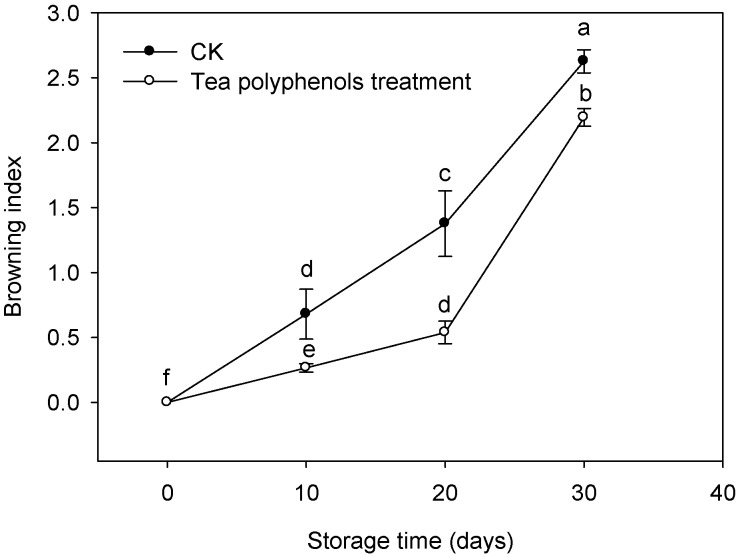
Effects of tea polyphenols on the browning index of litchi fruit during cold storage. Note that the values marked by different letters had significant differences at *p* < 0.05 level.

The browning index of litchi fruit gradually increased with prolonging storage time (Figure 2), which indicated that the fruit turned brown gradually.

In comparison with the control fruit, the fruits treated with tea polyphenols exhibited a lower pericarp browning index. The averages of control and polyphenols treatment groups were 2.19 and 2.63, respectively, after 30 days of cold storage.

### 2.2. The Changes in Contents of Total Soluble Solids and Ascorbic Acid

**Figure 3:**
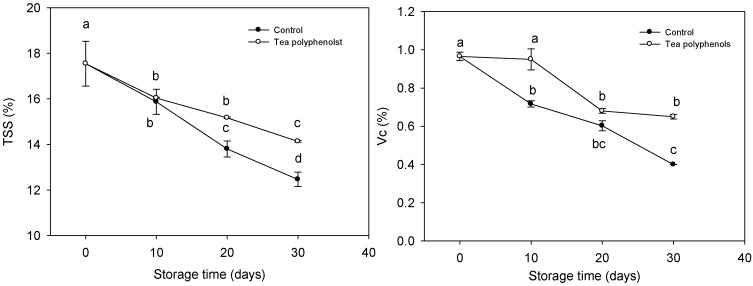
Effects of tea polyphenols on the contents of total soluble solids and ascorbic acid (Vc) in pulp of litchi fruit during cold storage. Note that the values marked by different letters had significant differences at *p* < 0.05 level.

The contents of total soluble solids (TSS) and ascorbic acid of litchi fruit decreased rapidly along with increasing storage time, while the control fruit exhibited a greater decrease in the contents of TSS and ascorbic acid (Figure 3). After 30 days of cold storage, the contents of ascorbic acid and TSS of the fruit treated with tea polyphenols were 62.5% and 13.4% higher than those of the control fruit.

### 2.3. The Changes in Relative Electric Leakage Rate and Malondialdehyde Contents

**Figure 4:**
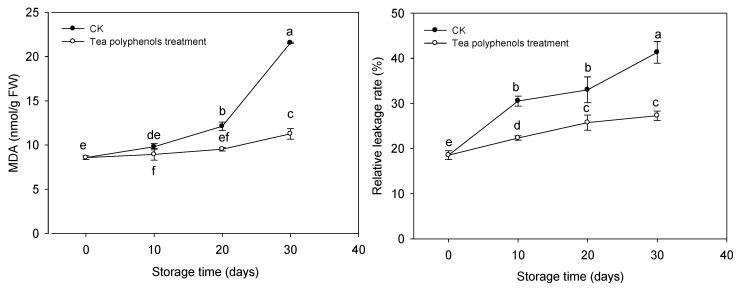
Effects of tea polyphenols on malondialdehyde content and relative leakage rate in pericarp tissues of litchi fruit during cold storage. Note that the values marked by different letters had significant differences at *p* <0.05 level.

Both the malondialdehyde (MDA) contents and relative leakage rates of litchi fruit were increased during storage (Figure 4).

The fruit treated with tea polyphenols exhibited significantly lower MDA content and relative leakage rate than the control fruit throughout this storage period. After 30 days of cold storage, the MDA content and relative leakage rate of the treated and control fruits were 21.56 and 11.27 nmol·g^−1^ FW, and 41.3% and 27.3%, respectively.

### 2.4. The Change in the Content of Reactive Oxygen Species

**Figure 5:**
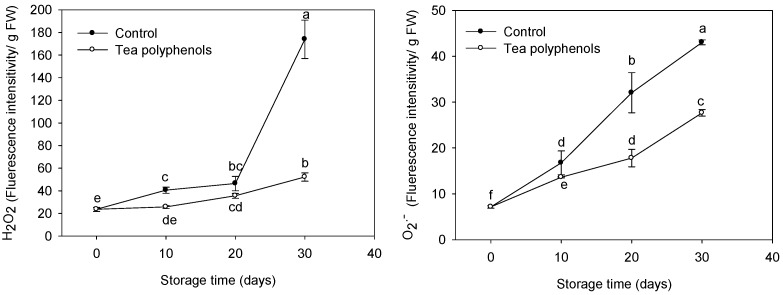
Effects of tea polyphenols on H_2_O_2_ content and O_2_^.−^ production rate in pericarp tissues of litchi fruit during cold storage. Note that the values marked by different letters had significant differences at *p* < 0.05 level.

The H_2_O_2_ contents and O_2_^.−^ production rate, as assayed by fluorescent probes, increased as the cold storage progressed (Figure 5). The treatment with tea polyphenols significantly reduced the increases in the H_2_O_2_ and O_2_^.−^ contents. After 30 days of cold storage, the H_2_O_2_ and O_2_^.−^ production rate of tea polyphenols-treated fruit were 30.0% and 64.3% of those of the control fruits.

### 2.5. The Changes in the Contents of Total Phenols and Anthocyanins

**Figure 6:**
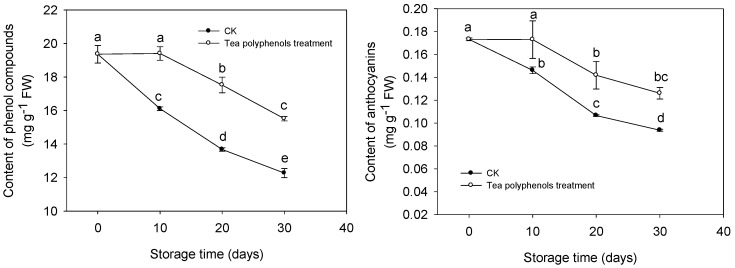
Effects of tea polyphenols on the contents of total phenols and anthocyanins in pericarp tissues of litchi fruit during cold storage. Note that the values marked by different letters had significant differences at *p* < 0.05 level.

As the storage time increased, the contents of total phenols and anthocyanins decreased rapidly. The treatment with tea polyphenols delayed the reduction in the contents of total phenols and anthocyanins (Figure 6).

After 30 days of cold storage, the content of total anthocyanins decreased by 45.9% and 27.2%, whereas the content of total phenols decreased by 36.6% and 19.9% for the control fruit and tea polyphenols-treated fruits, respectively.

### 2.6. The Change in the 1,1-Diphenyl-2-picrylhydrazyl Radical Scavenging Activity

**Figure 7:**
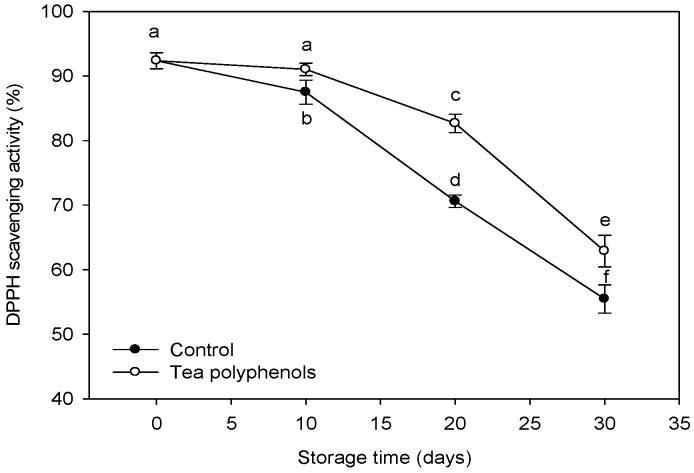
Effects of tea polyphenols on 1,1-diphenyl-2-picrylhydrazy scavenging activity of methanol extract of pericarp tissues of litchi fruit during cold storage. Note that the values marked by different letters had significant differences at *p* < 0.05 level.

The 1,1-diphenyl-2-picrylhydrazyl (DPPH) radical scavenging activity is associated with the contents of phenols and anthocyanins. As shown in Figure 7, the DPPH radical scavenging activity of the methanol extract of pericarp tissues of litchi fruit decreased gradually during storage. However, the fruit treated with tea polyphenols exhibited a significantly higher DPPH scavenging activity than the control fruit throughout this storage period.

### 2.7. The Changes in Activities of Antioxidative Enzymes

**Figure 8:**
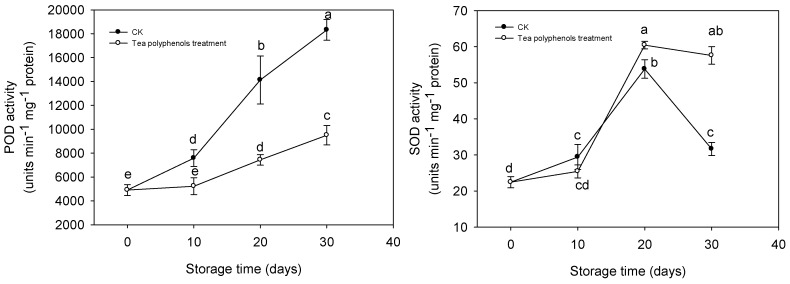
Effects of tea polyphenols on activities of superoxide dismutase and peroxidase in pericarp tissues of litchi fruit during cold storage. Note that the values marked by different letters had significant differences at *p* < 0.05 level.

POD activity of pericarp tissues of litchi fruit tended to increase gradually during storage (Figure 8). However, the treatment with tea polyphenols slowed down the increase in the POD activity of litchi fruit throughout this storage.

As shown in Figure 8, the SOD activity of litchi fruit increased and then decreased after 20 days of storage. At the later storage stage, the fruit treated with tea polyphenols exhibited a significantly higher SOD activity than the control fruit.

### 2.8. The Changes in Phenolic Compounds in Pericarp Tissues of Litchi Fruit during Cold Storage

**Figure 9:**
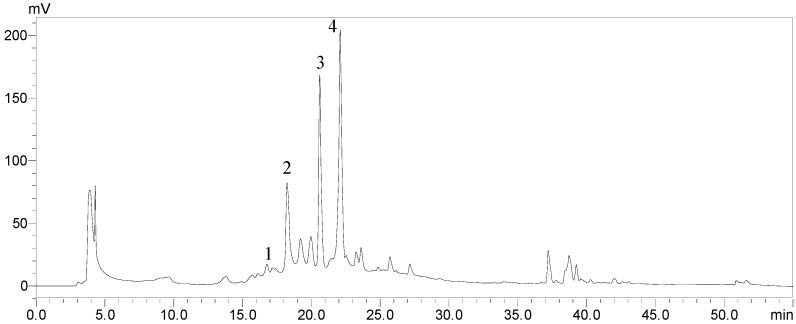
HPLC chromatogram of phenolic compounds in extract of litchi pericarp. Peak identification: (1) procyanidin B1; (2) catechin; (3) epicatechin; and (4) epicatechin 3-gallate.

Four phenolic compounds were identified in the pericarp extract of litchi fruit by HPLC (Figure 9). Among these four phenolic compounds, epicatechin was found to be present in the highest concentration. After 30 days of storage, the concentrations of all identified phenolic compounds in pericarp tissues of litchi fruit decreased (Table 1). The fruit treated with tea polyphenols exhibited significantly higher concentrations of catechin, epicatechin and epicatechin 3-gallatephenolic than the control fruit.

**Table 1:** The effects of tea polyphenols on the contents of various phenolic compounds (µg/g) in pericarp tissues of litchi fruit at harvest and after 30 days of storage. Corresponding means within a line between control and tea polyphenols treatment followed by the same letter are not significantly different at the 5% level. Note that the values marked by different letters had significant differences at *p* < 0.05 level.

Phenolic Compounds	Before Storage	Control	Tea Polyphenols
**Procyanidin B1**	0.038 ± 0.004 ^a^	0.034 ± 0.005 ^a,b^	0.027 ± 0.001 ^b^
**Catechin**	0.293 ± 0.008 ^a^	0.186 ± 0.002 ^c^	0.253 ± 0.003 ^b^
**Epicatechin**	2.532 ± 0.108 ^a^	1.229 ± 0.016 ^c^	1.913 ± 0.045 ^b^
**Epicatechin 3-gallate**	1.422 ± 0.062 ^a^	0.729 ± 0.007 ^c^	1.188 ± 0.047 ^b^

## 3. Discussion

Pericarp browning is a common and major problem of harvested litchi fruit during storage and transportation [10]. Some antioxidants were reported to inhibit enzymatic postharvest browning of fruits during storage and transportation. For example, it was reported that the combination of ascorbic acid and calcium chloride could reduce the enzymatic browning in sliced apple [11]. The application of anthocyanins extracted from seed coats of black beans markedly delayed pericarp browning of litchi fruit [4]. Similar inhibition in pericarp browning of litchi fruit was also achieved by using glutathione, a pivotal component of the glutathione-ascorbate cycle that functions by reducing poisonous hydrogen peroxide levels [8]. In addition, application of diphenylamine could effectively reduce peel browning of “Bartlett” pears induced by vibration, rolling or scuffing [12]. In this present study, the treatment with tea polyphenols significantly delayed the pericarp browning of the harvested litchi (Figure 2). To our knowledge, this is the first report that tea polyphenols exhibit a beneficial effect in delaying pericarp browning incidence in postharvest storage of litchi fruits. Furthermore, tea polyphenols extracted from tea leaves are a non-toxic food additive, suggesting the potential for commercial application as a pre-storage handling by dipping fruit in a 1% solution of tea polyphenols.

Although POD cannot directly oxidize litchi anthocyanins, the anthocyanins could be rapidly degraded by POD in the presence of H_2_O_2_ and some phenolics [13]. In the present study, Increased POD activity was consistent with browning index (Figure 8) but it was negatively related to total phenol or anthocyanin content (Figure 6). The application of tea polyphenols markedly inhibited the POD activity in pericarp tissues and maintained relatively higher levels of litchi fruit phenolic compounds and anthocyanins during storage (Table 1 and Figure 6), which may account for the delay of the pericarp browning of the fruit. The inhibitions of POD activity by exogenous ascorbic acid or citric acid were also reported in apple [11], melon [14], and lettuce [15], which was associated with reduced surface browning. The decrease in the contents of phenolics and anthocyanins may result from the oxidation by the accumulated ROS. Ruenroengklin *et al.* reported that the degradation of litchi anthocyanins by ROS in combination with the oxidation of endogenous phenolics by PPO could participate in browning reaction of harvested litchi fruit during storage [5]. The rapid increases in ROS contents was related to the decline of nonenzymatic oxidation defense capacity while the DPPH radical scavenging activity can be used as a reliable measure of nonenzymatic antioxidant activity both in plant tissues [16] and fish mince [17]. In this study, the decline in DPPH radical scavenging activity correlated to the increase in the ROS content of litchi pericarp (Figure 5 and Figure 7) and the application of tea polyphenols apparently enhanced the DPPH radical scavenging activity and reduced the increase in the ROS accumulations. Tea polyphenols are rich in catechins which mainly consist of epicatechin, epicatechin 3-gallate, epigallocatechin, epigallocatechin 3-gallate, catechin, and gallocatechin [18]. Catechins and their derivatives are the major bioactive compounds [19]. It has been reported that tea polyphenols have a strong ROS scavenging effect, much stronger than vitamin C and vitamin E [20]. Catechins were also reported to show the functions of protecting pigments and vitamins [21]. Thus, it was suggested that the strong antioxidant activity of tea polyphenols could be responsible for reducing lipid peroxidation of pericarp tissues of litchi fruit, which is possibly beneficial in scavenging free radicals, reducing ROS accumulation and then inhibiting the degradation of anthocyanins and the oxidation of endogenous phenolics, resulting in delayed pericarp browning of litchi fruit during storage.

The excessive ROS accumulation in combination with the decreases in antioxidant activities may also led to the membrane deterioration due to lipid peroxidation. A great deal of evidence shows that lipid peroxidation leads to a loss of membrane integrity resulting in damage of compartmentation between enzymes and their substrates and, thereby, may enhance enzymatic browning of litchi fruit [1]. In this present investigation, applications of tea polyphenols resulted in a lower concentration of MDA (Figure 4), the main product of lipid peroxidation, compared with control fruit, indicating that lipid peroxidation of the treated litchi fruit was reduced. Thus, it was suggested that enhanced antioxidant activity by the treatment with tea polyphenols could be responsible for reducing lipid peroxidation of litchi fruit.

## 4. Experimental Section

### 4.1. Plant Materials and Treatments

Fruit of litchi (*Litchi chinensis* Sonn.) cv. Huaizhi at commercial maturity (a full red skin color) were obtained from an orchard in Guangzhou, China. Fruits with uniform shape, color and size were selected and then used for the study. Tea polyphenols, a commercial product with a purity of 97%, were obtained from Rongbo Chemical Co. Ltd (Huzhou, China). In the preliminary test, application of 1% tea polyphenols exhibited an optimum effect on the storage life extension of litchi fruit. In this study, the fruit were dipped in a solution of 1.0% tea polyphenols for 5 min. Fruit treated with distilled water were used as control. After treatment, the fruit were air-dried for 2 h at 25 °C before being packed into plastic punnets (20 fruits/punnet), overwrapped with plastic films and stored at 4 °C and 70%–80% relative humidity.

### 4.2. Browning Index Assessment

Browning was assessed by the extent of the browned area on each fruit’s pericarp [22], using the following scale: 0, no browning; 1, <1/4 browning; 2, 1/4–1/2 browning; 3, 1/2–3/4 browning; and 4, >3/4 browning. The browning index was calculated as the following formula: Σ (browning scale × percentage of fruit within each class).

### 4.3. Determinations of Contents of Ascorbic Acid and Total Soluble Solids

The contents of ascorbic acid and total soluble solids (TSS) of litchi fruit were analyzed after 0, 10, 20 and 30 days of storage. Pulp tissues (20 g) from 10 fruits were homogenized in a grinder and then centrifuged at 15,000*× g* for 20 min. The supernatant was collected for analyses of content of ascorbic acid by 2,6-dichlorophenol indophenol titration [23]; and total soluble solids using a hand refractometer (J1-3A, Guangdong Scientific Instruments, Guangzhou, China).

### 4.4. Analyses of Membrane Permeability and Lipid Peroxidation

Membrane permeability, expressed by relative electrolyte leakage rate, was determined by the method described in [24]. Peel discs were removed with a 5 mm in diameter cork borer from the equatorial region of 30 fruit. Fifty discs were rinsed twice in distilled water and then incubated in 30 mL of 0.3 M mannitol solution at 25 °C with shaking for 30 min. Electrolyte leakage was determined with a conductivity meter (Model DDS-11A, Shanghai Scientific Instruments, Shanghai, China). Another batch of discs was boiled for 15 min in 25 mL of 0.3 M mannitol in distilled water and then cooled to 25 °C to assess total electrolytes. Relative leakage was expressed as a proportion total electrolyte leakage.

Lipid peroxidation was estimated by the content of malondialdehyde (MDA) according to the method described by [25]. Pericarp tissues (5 g) from 30 litchi fruits were homogenized with 25 mL of 50 g/L trichloroacetic acid (TCA) and then centrifuged for 10 min at 4000× *g*. The collected supernatant (1 mL) was mixed with 3 mL of 0.5% thiobarbituric acid (TBA) dissolved previously in 10% trichloroacetic acid. The reaction mixture solution was heat-treated for 20 min at 95 °C, cooled quickly, and then centrifuged for 10 min at 10,000× *g* to clarify the supernatant. Absorbance was measured at 532 nm and corrected for nonspecific turbidity by subtracting the absorbance at 600 nm. Blanks containing TCA instead of TBA solution were used as a control. MDA concentration was calculated with an extinction coefficient of 1.55 mM^−1^·cm^−1^.

### 4.5. Measurements of H_2_O_2_ and O_2_^.−^ Contents

The H_2_O_2_ and O_2_^.−^ contents were determined using the fluorescent probes 5-(and 6)-carboxy-2,7-dichlorodihydrofluorescein diacetate (H_2_DCFDA, Molecular Probes, Eugene, OR, USA) and dihydroethidium (DHE, Molecular Probes) [26,27]. Stocks of H_2_DCFDA and DHE were diluted carefully with dimethyl sulfoxide (DMSO) to a concentration of 5 mM and then stored in dark at −80 °C. Pericarp tissues (0.2 g) from 10 fruits were homogenized with 1.5 mL of 0.05 mol·L^−1^ Tris-HCl buffer (pH 7.0) and then centrifuged at 12,000× *g* for 20 min. The collected supernatant (0.4 mL) was mixed with 3.6 mL of 0.05 mol·L^−1^ Tris-HCl buffer (pH 7.0), and then labeled with 10 µM DHE or H_2_DCFDA for 40 min at 37 °C. Samples were analyzed for H_2_O_2_ and O_2_^.−^ contents using a FACScan flow cytometer (Becton Dickinson Immunocytometry Systems, Inc., Mountain View, CA, USA) (excitation 488 nm with emission 585 nm for DHE and emission 530 nm for H_2_DCFDA band-pass filters).

### 4.6. Determinations of Activities of Superoxide Dismutase and Peroxidase

Pericarp tissues (5 g) from 10 fruits were homogenised in 20 mL 0.05 M potassium phosphate buffer (pH 7.0) contained 0.5 g of polyvinylpyrrolidone (insoluble). After filtration through a cotton cloth, the filtrate was centrifuged for 20 min at 20,000× *g* and 4 °C. The supernatant was collected and used as the crude enzyme extract. Protein content was determined according to the method of [28] with bovine serum as the standard.

Superoxide dismutase (SOD) activity was assayed by measuring its ability to inhibit the photochemical reduction of nitroblue tetrazolium (NBT) by the method of [29]. One unit of SOD activity was defined as the amount of enzyme required to inhibit 50% of the reduction of NBT per mg protein as monitored at 560 nm.

Peroxidase (POD) activity, using guaiacol as a substrate, was assayed by the method described by [13]. The reaction mixture (3 mL) contained 50 µL of enzyme extract, 2.75 mL of 50 mM phosphate buffer (pH 7.0), 0.1 mL of 1% H_2_O_2_ and 0.1 mL of 4% guaiacol. The increase in absorbance at 470 nm caused by the guaiacol oxidation was recorded for 2 min. One unit of enzyme activity was defined as the amount that caused a change of 0.01 in absorbance per minute.

### 4.7. Determination of 1,1-Diphenyl-2-picrylhydrazyl Radical Scavenging Activity

The 1,1-diphenyl-2-picryl hydrazyl (DPPH) radical scavenging activity was determined according to the method described by [4], with some modifications. Briefly, pericarp tissues (5 g) from 10 fruits were ground in liquid nitrogen using a pestle and bowl, and then extracted with 30 mL of methanol containing 0.2 g sodium metabisulphite for 30 min. After filtrating through four layers of cheesecloth and centrifugation 20 min at 20,000× *g*, the supernatant was collected and used for determination of DPPH radical scavenging activity. The methanol extract (100 µL) was mixed with 2.9 mL of 0.1 mM DPPH-methanol solution and then incubated for 30 min in dark at 25 °C. The DPPH radical-scavenging activity was determined by measuring the absorbance at 517 nm using a spectrophotometer. The antioxidant activity was expressed as a percentage of scavenging DPPH radicals and calculated using the following formula: (Control OD − Sample OD)/Control OD × 100.

### 4.8. Determinations of Contents of Total Phenols and Anthocyanins 

A total of 5 g of peel was homogenised and extracted by 100 mL HCl-methanol (v:v = 1:99) at room temperature and extracted for 30 min. After the filtration the total phenolic contents were measured by the Folin-Ciocalteu reaction method and then expressed as gallic acid equivalents [30] while anthocyanin contents in pericarp tissues were determined using the pH differential method [31]. Results were expressed as milligrams of cyanidin-3-glucoside equivalents per g fresh weight.

### 4.9. Analyses of Phenolic Compounds

Phenolic compounds were extracted and purified according to the methods of [32]. Fresh pericarp tissues (10 g) were homogenised with cold 65% ethanol (50 mL) containing 0.5% sodium metabisulphite in an ice bath and kept overnight. The homogenate was filtered through four layers of cheesecloth and then the filtrate was centrifuged for 20 min at 10,000× *g*. The supernatant was collected and then filtered through Ø 0.45 mm filters. A 20-mL aliquot of the filtrate was injected directly into the high-performance liquid chromatography (HPLC) system (Shimadzu, Kyoto, Japan). HPLC analysis was performed on an LC-20 AT system (Shimadzu) using a Shim-pack UP-ODS C18 column (4.6 × 250 mm) and a diode array detector (Rheodyne, Cotati, CA, USA). The samples were eluted with a gradient system consisting of solvent A (2% acetic acid) and solvent B (acetonitrile/methanol = 10:15), with a flow rate of 1 mL/min. The temperature of the column was maintained at 25 °C, while the monitoring wavelength was 280 nm. The identification of the phenolic compounds was based on a combination of retention time and spectral characteristics matching with standard compound from Sigma-Aldrich Company (St. Louis, MO, USA). The quantification was made via a calibration with standard compound using the external standard method.

### 4.10. Statistical Analysis

The experiments were arranged in completely randomized design. For each parameter measurement, triplicate assays were conducted. The Duncan’s Multiple Range Test (SPSS 13.0 statistical software, SPSS Inc., Chicago, IL, USA) was used to determine significant differences. Data were presented as the means ± standard errors (SE) of three replicate determinations. The least significant differences (*p* = 0.05) were calculated for mean separation.

## 5. Conclusions

In conclusion, no obvious weight loss was observed in a storage period of 30 days, which may be due to tight packaging with plastic bags. The treatment with tea polyphenols inhibited markedly POD activity in association with high contents of total anthocyanins and phenolics, and low ROS accumulation that resulted in reduced lipid peroxidation and partial maintenance of membrane integrity, which may be responsible for delayed pericarp browning of litchi fruit during storage. Thus, tea polyphenols are potentially useful in postharvest handling of litchi fruit to inhibit pericarp browning and maintain high quality during storage and transportation.
